# Morphological Study of Variations of the Human Cadaveric Liver and Its Clinical Implications

**DOI:** 10.7759/cureus.35507

**Published:** 2023-02-26

**Authors:** Kumar Sambhav, Hare Krishna, Shilpi Gupta Dixit, Surajit Ghatak

**Affiliations:** 1 Anatomy, All India Institute of Medical Sciences, Jodhpur, IND

**Keywords:** hepatic morphology, caudate lobe, quadrate lobe, accessory fissures, anatomical variations, liver

## Abstract

Introduction

With increasing dependence on laparoscopic procedures, precise knowledge of external variations of the liver is essential for good surgical and interventional outcomes, preventing imaging misdiagnosis, and curtailing complications. The present study aims to evaluate the gross anatomical variations of the liver.

Materials and Methods

The 40 adult cadaveric livers of age 60-80 years were removed during the routine dissection for undergraduate medical students and examined for morphological variations in the form of size, shape, and fissures.

Results

Accessory fissures were observed on the caudate lobe (CL) in 23 (57.5%), on the quadrate lobe (QL) in seven (17.5%), on the right lobe (RL) in 29 (72.5%), and on the left lobe (LL) in 12 (30%) specimens. Netter’s Type 2, Type 4, Type 5, Type 6, and Type 7 liver were observed in four (10%), seven (17.5%), one (2.5%), three (7.5%), and three (7.5%) specimens respectively. The most common shapes of the CL and QL were rectangular in 16 (40%) and quadrangular in 10 (25%) specimens respectively. Pons hepatis were seen in three (7.5%) specimens. The mean length (cm) of RL and LL were 17.75 ±3.09 and 16.9±3.69 respectively, whereas the mean transverse diameter (TD) (cm) of RL and LL were 7.98±1.20 and 7.85±1.58 respectively. The mean length and TD (cm) of CL were 5.62±1.67 and 2.48±1.00 respectively. The mean length and TD (cm) of the QL were 6.00±1.51 and 2.81±0.83 respectively.

Conclusion

Precise knowledge of these variations would be helpful for surgeons in planning and performing surgical procedures and for anatomists.

## Introduction

The segmental liver, although it has gathered immense attention with revisions over time, external variations have acquired consideration only recently and require in-depth study in view of increased interventions and hepatobiliary surgeries. The anatomy of the liver is complex and challenging owing to its widespread morphological variations which present as congenital or acquired, occurring in the form of varied shapes of lobes, fissures, congenital anomalies encompassing agenesis, atrophy or hypoplasia, accessory lobes and fissures, and undue size of a particular lobe, all due to interrupted development at a precise embryonic stage [1,2]. Whereas diaphragmatic, ligamentous anomalies and changes induced by various organs in close relation during lifetime could be the cause for acquired variations. Most of the accessory fissures disappear during the liver reformation in the postnatal period but some can persist for life [3]. The localization of the major fissures contributes to the interpretation of lobar anatomy and localizing lesions [4]. Also, metastatic tumors could be lodged in these spaces mimicking focal lesions [5]. The morphological changes in cirrhosis and non-cirrhotic liver disease include atrophy of the right lobe (RL) and segment 4 with hypertrophy of the left lobe (LL) and expansion of the gall bladder (GB) fossa [6]. Therefore, with increasing dependence on radiological imaging, and laparoscopic procedures, precise knowledge of external variations of the liver is indispensable for favourable surgical and gastroenterological interventional results, preventing imaging misdiagnosis and curtailing unnecessary surgical complications [2]. The objective of the current study is to evaluate the external anatomical variations of the liver and their clinical implications.

## Materials and methods

This observational study was conducted on 40 adult human livers from embalmed male and female donors of age 60-80 years after ethics approval from the Institutional Ethics Committee (AIIMS/IEC/2021/3758) in the Department of Anatomy, All India Institute of Medical Sciences, Jodhpur, India. The livers were removed en bloc along with the intrahepatic part of the inferior vena cava (IVC) during routine dissection for medical undergraduate teaching and preserved in 10% formalin. Livers with evident pathological conditions, metastatic disease, damage, and surgical resections were excluded from the study. The gross variations of lobes of the liver such as variations in dimensions, shape, processes, atrophy, agenesis, the incidence of pons hepatis, accessory lobes, and fissures were photographed and the morphological variations of the liver were categorized on the basis of Netter’s classification [2]. The measurements of maximum length and width (transverse diameter [TD]) of the RL, LL, caudate lobe (CL) and quadrate lobe (QL) along with the length of accessory fissures on the RL and LL were also measured using a digital vernier caliper and a measuring tape where caliper measurements were not feasible. To determine the TD of the CL, the midpoint of the hepatic part of IVC was taken as a reference point for the right lateral margin. Similarly, the same reference point was used for the TD of the RL of the liver. The left margin of the fissure for ligamentum venosum (or medial limit of LL) was considered a reference point for the TD of the LL. The CL/RL ratio (ratio of TD of CL/TD of the RL) was determined. The length of the CL was calculated from its lower border above the porta hepatis and portal vein. The inferior limit of the hepatic part of IVC was determined at the posterior border of the caudate process (CP) of CL. Similarly, the length and TD of the QL of the liver were recorded. The number and percentage of each morphological feature were recorded. The numerical data were summarized as arithmetic mean±standard deviation (SD).

## Results

The various morphological variations including Netter’s classification, the shape of CL, QL, and papillary process (PP) along with hypertrophied and hypoplastic lobes, accessory fissures and notches, the incidence of the accessory lobe, and pons hepatis (PH) were all observed and recorded.

Gross variations of the liver based on Netter’s classification

**Figure 1:**
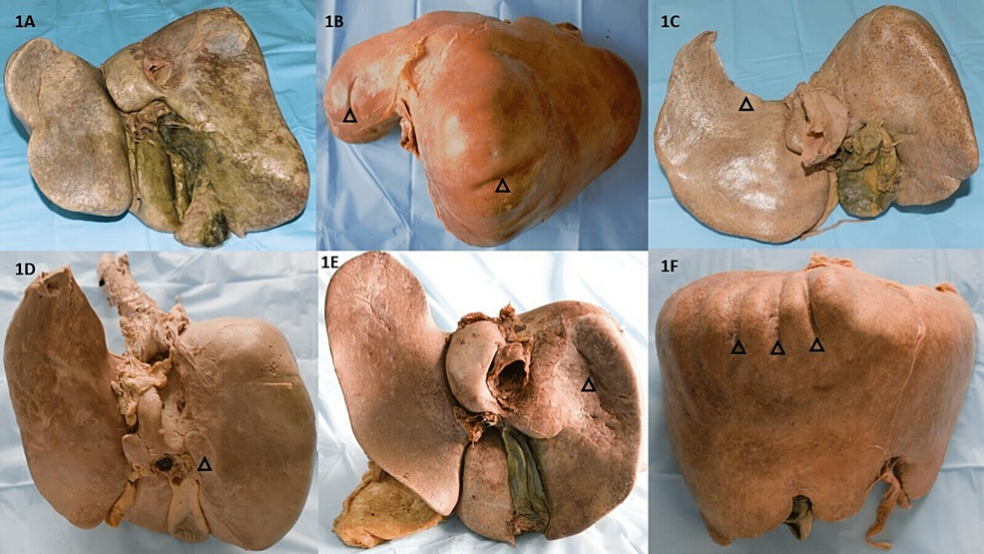
Livers categorised on the basis of Netter’s classification 1A: Netter’s Type 1 liver (normal characteristics); 1B: Netter’s Type 2 liver (small LL with deep costal impressions); 1C: Netter’s Type 4 liver (transverse saddle-like liver with relatively large LL); 1D: Netter’s Type 5 liver (tongue-like process of RL); 1E: Netter’s Type 6 liver (very deep renal impression); 1F: Netter’s Type 7 liver (diaphragmatic grooves on the surface). RL: right lobe, LL: left lobe

Based on Netter’s classification [2], Type 1 (normal) was observed in 22 (55%) specimens, Type 2 in four (10%), Type 7 in four (17.5%), and Riedel’s lobe was observed in only one (2.5%) specimen (Table 1). No liver with complete atrophy of the LL (Type 3) was observed. This classification mentions acquired morphological changes in the form of Type 6 -deep renal impression and corset impression. The various livers belonging to various types based on Netter’s classification are depicted in Figures 1A-1F.

**Table 1:** Prevalence based on gross variations of the liver in accordance with Netter’s anatomical classification [2]

	Gross Description	n=40
Type 1	Normal	22 (55%)
Type 2	Very small left lobe, deep costal impressions	4 (10%)
Type 3	Complete atrophy of the left lobe	-
Type 4	Transverse saddle like liver, relatively large left liver	7 (17.5%)
Type 5	Tongue like process of the Right lobe	1 (2.5%)
Type 6	Very deep renal impression & corset constriction	3 (7.5%)
Type 7	Diaphragmatic grooves on the surface	3 (7.5%)

Morphological variations of the caudate lobe

**Figure 2:**
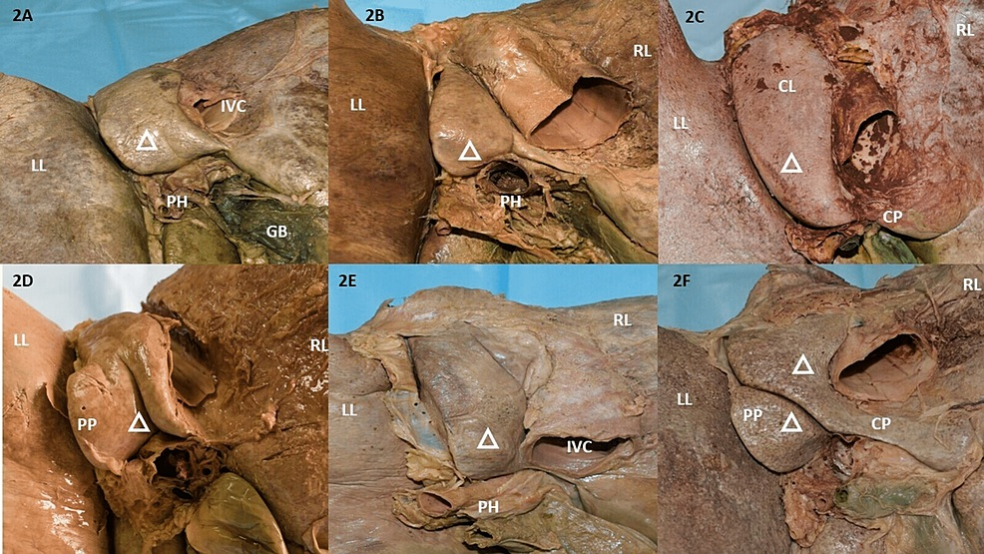
Morphological variations of the caudate lobe. 2A: Rectangular caudate lobe; 2B: Pear/pyriform caudate lobe with just discernible CP; 2C: Inverted pyriform caudate lobe, normal CP, absent PP; 2D: Bicornuate caudate lobe with prominent PP and prominent CP; 2E: Irregularly shaped caudate lobe, CP overlapped by IVC can be termed as just discernible; 2F: Bilobed CL, complete transverse fissure dividing CP into a detached PP which can be also termed as an accessory lobe and a prominent long CP. GB: gall bladder, RL: right lobe, LL: left lobe, PH: pons hepatis, CP: caudate process, PP: papillary process, IVC: inferior vena cava, CL: caudate lobe

All variant types are depicted in Figures 2A-2F and tabulated in Table 2. The most common shape encountered was rectangular (40%) with irregular and elongated shapes being the least common (7.5% each) (Figures 2A, 2E). Bilobed CL, a rather rare observation was also observed in 7.5% of specimens (Figure 2F). Normal-sized CLs were observed in 72.5% of livers (Figure 2A). There was evidence of CL hypoplasia in 15% of livers. All livers presented with CPs and PPs with varying sizes except three specimens, one of which showed absent CP and PP. The other presented with a discernible CP but no PP and the third with an absent PP. No evidence of aplasia was noted.

**Table 2:** Morphological variations of the caudate lobe of the liver

Variation Category		Number (n=40)/Percentage
Rectangular	16 (40%)	
Bicornuate	6 (15%)	
Pear/Pyriform	5 (12.5%)	
Inverted Pyriform	4 (10%)	
Irregular	3 (7.5%)	
Elongated	3 (7.5%)	
Bilobed (complete fissure)	3 (7.5%)	
Pons Hepatis	1 (2.5%)	
Caudate Lobe Underdeveloped/hypoplastic	6 (15%)	
Prominent	5 (12.5%)	
Papillary Process Normal	17 (42.5%)	
Prominent	13 (32.5%)	
Grossly Prominent	7 (17.5%)	
Absent	3 (7.5%)	
Caudate Process Normal	30 (75%)	
Prominent	6 (15%)	
Just discernable	3 (7.5%)	
Absent	1 (2.5%)	

Morphological variations of the quadrate lobe

**Figure 3:**
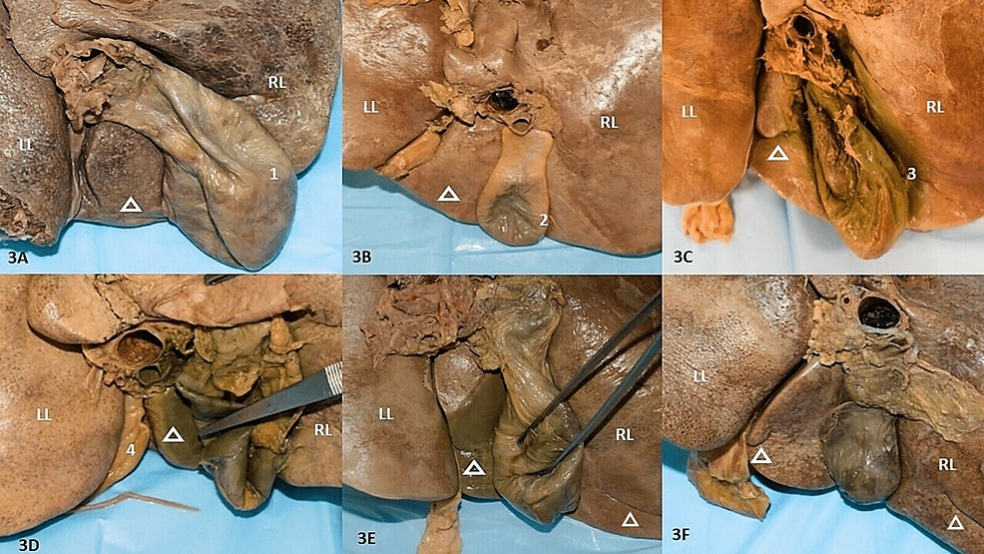
Morphological variations of the quadrate lobe. 3A: Quadrangular QL, 1- extensive GB fossa; 3B: Rectangular QL, 2- embedded GB fossa; 3C: Bilobed QL with a horizontal fissure, 3- deep GB fossa; 3D: Narrow QL overlapped by GB, 4- widened fissure for ligamentum teres hepatis; 3E: Elongated QL with incomplete horizontal fissure and accessory horizontal fissure above the inferior border; 3F: Small projection presenting as mini accessory lobe from triangular shaped QL and accessory horizontal fissure above the inferior border. GB: gall bladder, QL: quadrate lobe, RL: right lobe, LL: left lobe

All variants are depicted in Figures 3A-3F and tabulated in Table 3. The most common shape observed was quadrangular in 25% followed by rectangular in 17.5% of specimens (Figures 3A, 3B). Bilobed QL was observed in 10% of livers (Figure 3C). The least common shape was elongated in 5% of specimens (Figure 3E). Mini accessory lobes accounted for 5% of all specimens (Figure 3F). Irregular and rectangular shapes were observed in 12.5% and 15% of specimens respectively.

**Table 3:** Morphological variations of the quadrate lobe of the liver

Variation Category	Number (n=40)/ Percentage
Quadrangular	10 (25%)
Triangular	7 (17.5%)
Rectangular	6 (15%)
Irregular	5 (12.5%)
Narrow	3 (7.5%)
Elongated	2 (5%)
Pons Hepatis	2 (5%)
Bilobed	2 (5%)
Absent	1 (2.5%)
Accessory Fissures	7 (17.5%)
Mini Accessory lobe	2 (5%)
Fossa for Gall bladder – deep	20 (50%)
extensive	10 (25%)
embedded	7 (17.5%)
absent	3 (7.5%)

Fissures in the caudate lobe

**Figure 4:**
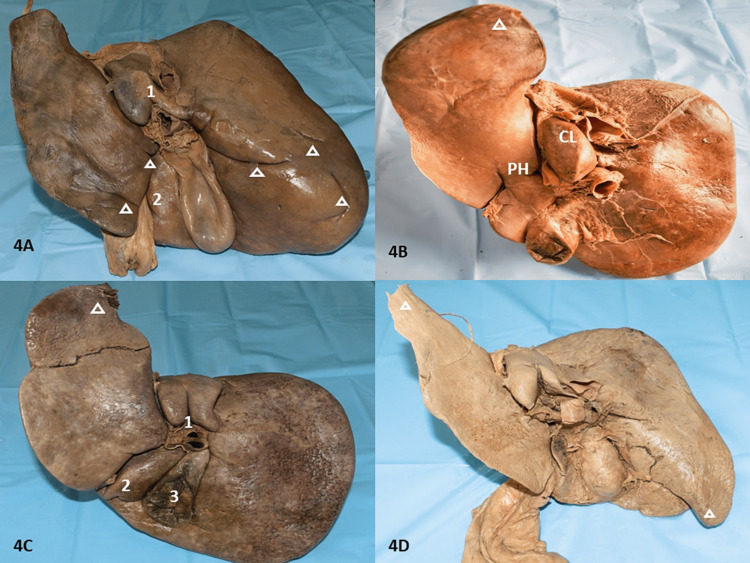
Depicting accessory fissures in various lobes of the liver 4A: Three accessory fissures on RL. The largest 5.8 cm long prominent oblique fissure, crescent shaped fissure and smallest oblique fissure. Oblique fissure arising from the inferior surface of CL dividing larger PP and a normal CP with two accessory fissures on LL meeting the FLT; 4B: Prominent CP with absent PP and CP. PH in the upper two-thirds for FLT. Elongated LL (Netter’s Type 4 liver); 4C: 1-vertical fissure arising from the center of the inferior margin of CL and bifurcating PP and CP, 2- Tongue-like projection posing as mini accessory lobe from irregular QL, 3- Embedded GB; 4D: Reidel’s lobe with the accessory process from LL. RL: right lobe, CL: caudate lobe, PP: papillary process, CP: caudate process, LL: left lobe, FLT: fissure for ligamentum teres, PH: pons hepatis, IVC: inferior vena cava, QL: quadrate lobe, GB: gall bladder

In addition to variations in the appearance of CL, the fissures in CL accounted for 57.5% (23 specimens) varying in length and prominence. Of all the fissures, vertical fissures comprised the maximum 71.4% (15 fissures) (Figure 2D), followed by 19% complete oblique fissures (Figure 2F), one (4.8%) complete transverse, and one (4.8%) incomplete oblique fissure (Figure 4A). All vertical fissures distinctively separated the CP from the CL. The complete oblique fissures divided the CL and the PP in their entirety. One specimen presented with a prominent CL with no fissures and was devoid of both PP and CP. This was also associated with a large LL (Type 4 liver) (Figure 4B). In 50% of specimens, fissures were observed to originate from the inferior border of the CL. 6.6% of such fissures arose from the centre of the inferior margin of CL and 53.3% of fissures arose slightly away from the middle of the inferior margin (Figure 4C). The remaining 20.1% of fissures arose from the inferior margin but adjacent to the IVC. Reidel’s lobe with the accessory process from LL was observed in one specimen (Figure 4D). All livers that presented with an accessory process of LL (four livers) were observed to have both CP and PP, whereas, most specimens presented with normal CP and PP. The dimensions of the fissures on the CL were not recorded. All findings are tabulated in Table 2.

Fissures in the quadrate lobe

**Figure 5:**
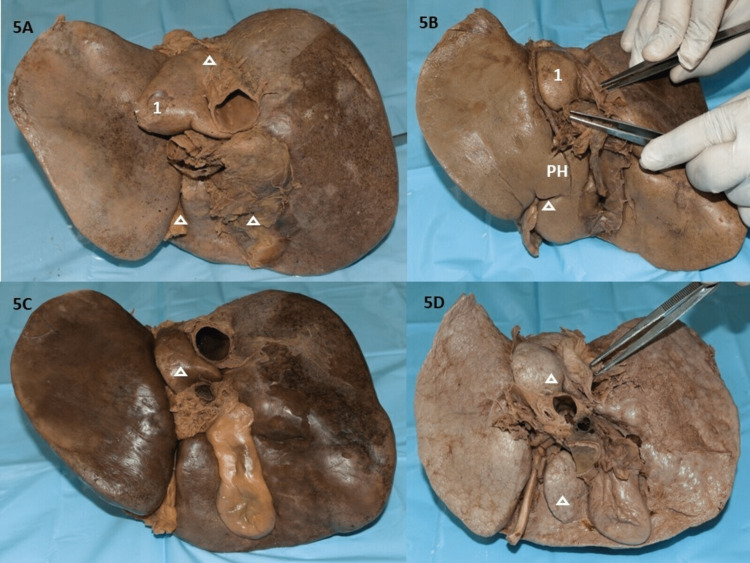
Variant papillary process and coexistent findings. 5A: Grossly prominent PP with PH encapsulating IVC with small irregular QL and absent GB; 5B: PH in upper two-thirds of fissure for ligamentum teres hepatis (FLT) extending as an incomplete oblique fissure; 5C: Prominent PP, normal CP with embedded GB; 5D: Grossly prominent PP with normal CP, curved fissure in quadrangular QL. PP: papillary process, IVC: inferior vena cava, QL: quadrate lobe, GB: gall bladder, PH: pons hepatis, CP: caudate process

The QL was observed to have seven (17.5%) fissures in all specimens (Figures 3, 4, 5). Amongst them, two had horizontal fissures that divided the lobe into superior and inferior QL. In one such specimen the superior lobe could be lifted (Figure 3C). Three specimens presented with curved fissures and two specimens with oblique fissures (Figures 4A, 5A, 5B, 5D). In two specimens, the PH was observed in the upper two-thirds of the fissure for ligamentum teres hepatis which continued a fissure to partially divide into superior and inferior QL (Figures 4B, 5B). In one irregular-shaped QL, a mini accessory lobe, appearing as a tongue-like projection was observed (Figure 4C). All observations have been tabulated in Table 3.

Accessory fissures in the right and left lobes

**Figure 6:**
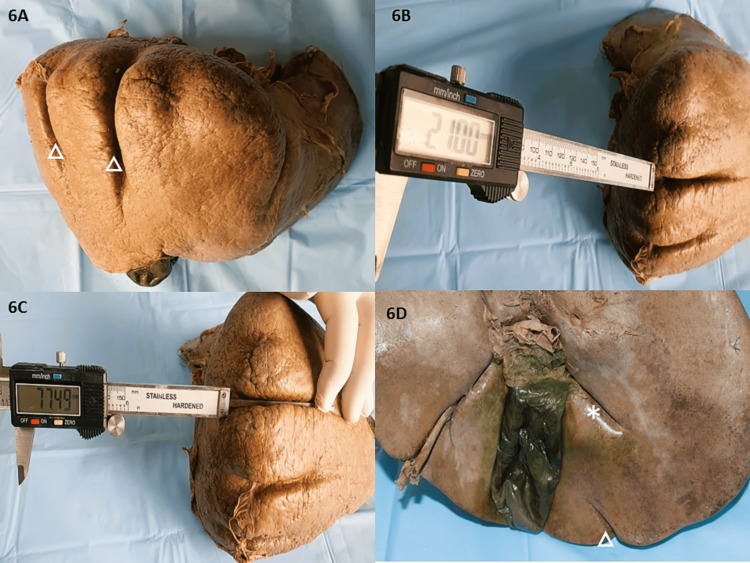
Depicting deep dimensions of Zahn's grooves and fissure of Gans 6A: Very deep and prominent diaphragmatic grooves/Zahn’s grooves (three in number) on the superior and anterior surface of the liver; 6B: Depth measurement of the most prominent diaphragmatic groove (2.1cm); 6C: Length of the longest diaphragmatic groove (7.75cm); 6D: Deep fissure arising from inferior border coursing parallel to fissure of Gans above (star marked).

The proportion of accessory fissures was substantially higher on the RL vis-à-vis the LL. The average length ranged from 1.8-5.8cm varying in prominence. These accessory fissures were observed in 29 RLs (72.5%) and 12 LLs (30%). One liver specimen was seen to be devoid of any such fissure whatsoever. Three specimens showed deep diaphragmatic grooves on the superior surface of the liver (Netter’s Type 7), one of which was more remarkable with three very deep and prominent grooves. The longest and deepest grooves measured 7.75cm in length and 2.1cm in depth respectively (Figures 6A-6C). A 3.2cm deep fissure was observed 4cm lateral to the cystic notch arising from the inferior border coursing almost parallel to the fissure of Gans in one liver (Figure 6D). 

In one specimen, the RL presented three fissures, wherein a 5.8cm long prominent oblique fissure from the neck of the gall bladder demarcated the CP above and the duodenal impression below ending below the renal impression and with the colic impression below (Figure 4A). This could be remarked as a vascular fissure or Rouviere’s sulcus/fissure of Gans. Such a fissure was not found in the LL. The second crescent-shaped fissure coursed towards the meeting point of the right lateral and inferior border. The smallest oblique fissure was observed at the inferior margin of renal impression within the attachment of the coronary ligament (Figure 4A). The same liver had two superficial fissures on the LL, with the smaller superior fissure in approximation to porta hepatis along with the larger fissure observed encroaching the fissure for ligamentum teres hepatis. Two livers presented with similar 4cm and 4.7cm long prominent oblique fissures beginning at the neck of the gall bladder and continuing below the CP which can be remarked as vascular fissures. Three livers were observed to have an almost horizontal fissure 2cm above the inferior margin running for a length of 5-5.8cm and approaching the meeting point of the right and inferior borders. Their course can be appreciated in Figures 3E, 3F.

Pons hepatis

PH was observed in the upper two-thirds for FLT in two specimens (Table 3, Figures 4B, 5B) and another PH encapsulating the IVC was observed in one specimen (Table 2, Figure 5A). 

Accessory notches

All livers in our study were observed with an umbilical notch meant for the falciform ligament and a notch for the fundus of the gall bladder. A total of five (12.5%) accessory notches were observed. One accessory notch was seen near the meeting point of the right and inferior borders of the RL and one on the right inferior border (Figure 1C). Two accessory notches were observed on the LL. One was midway on the left lateral border of the LL (Figure 1A) and the other was slightly above the midpoint of the left lateral border (Figure 4C).

**Figure 7:**
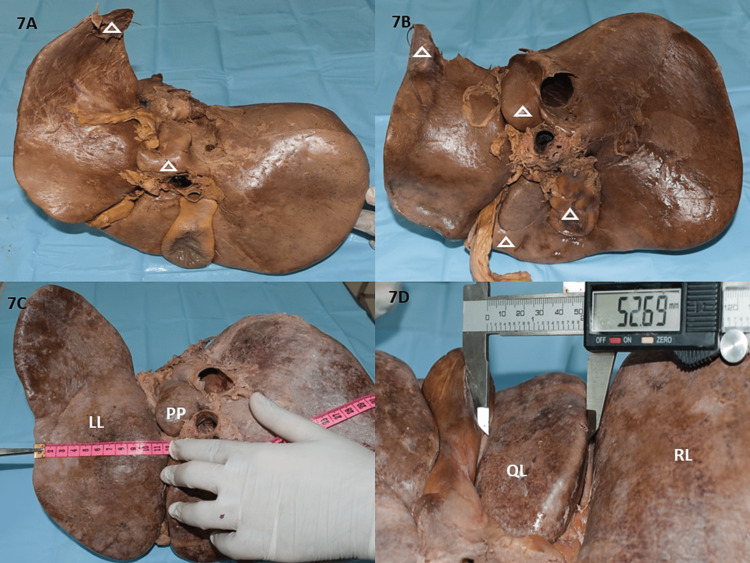
Depicting accessory process and maximal measurements by Vernier caliper and measuring tape 7A: Accessory process from LL along with irregularly shaped CL presenting as normal PP and CP, Quadrangular QL; 7B: Grossly prominent PP, embedded GB with curved fissure on QL with the accessory process on LL; 7C: Maximum TD of LL recorded using a measuring tape depicting a grossly prominent PP; 7D: Maximum TD of QL measured by Vernier caliper. RL: right lobe, LL: left lobe, CL: caudate lobe, PP: papillary process, CP: caudate process, QL: quadrate lobe, GB: gall bladder, TD: transverse diameter

The accessory process from LL are depicted in Figures 7A, 7B. Fossa for GB presented as deep (50%), extensive (25%), embedded (17.5%), and was observed to be absent in 7.5% of specimens (Figures 3A, 3B, 5C, 7B). The length of the RLs and LLs ranged between 10.2-26cm (Mean±SD =17.75±3.09) and 7.8-27cm (Mean±SD=16.9±3.69) respectively. Whereas the width (TD) of the RLs and LLs (Figure 7C) ranged between 5.6-11.5cm (Mean±SD=7.98± 1.20) and 4.6-10.7cm (Mean±SD =7.85±1.58) respectively (Table 4). The TD of the CL ranged between 1.2-5.8cm (Mean±SD=2.48 ± 1.00), which was utilized to describe the size of the CL and in most shapes of the CL as observed in this study was at a higher level than the main portal vein. The TD of QL (Figure 7D) ranged between 1.5-5.26cm (Mean±SD =2.81 ± 0.83).

**Table 4:** Gross measurements of various dimensions of lobes of the liver

Measurement categories	Range (cm)	Mean ± SD
Length of the left lobe	7.8-27	16.9 ± 3.69
Transverse diameter of the left lobe (TD)	4.6-10.7	7.85 ± 1.58
Length of the right lobe	10.2-26	17.75 ± 3.09
Transverse diameter of the right lobe (TD)	5.6-11.5	7.98 ± 1.20
Length of the caudate lobe (CL)	2.3-9	5.62 ± 1.67
Transverse diameter (TD) of CL	1.2-5.8	2.48 ± 1.00
Length of the quadrate lobe (QL)	2.8-10.3	6.00 ± 1.51
Transverse diameter of QL	1.5-5.26	2.81 ± 0.83
TD-CL/TD-Right lobe (CL/RL) Ratio	0.15-0.67	0.32 ± 0.13

## Discussion

In this present study, livers confirming all types of Netter’s classification were observed except for Type 3, whereas Type 7 livers were documented in 7.5% of livers which hasn’t been observed by many studies. The varying shapes are significant criteria leading to misdiagnoses and unfavorable surgical outcomes [4]. Clinical challenges can occur with infrequently reported accessory lobes primarily due to being asymptomatic or the presence of Rouviere’s sulcus which has been rarely mentioned by authors [7,8]. Both of these have been reported in this study. The human liver presents with an array of congenital variations primarily due to faulty or disproportionate development [3,9], whereas occasionally they present with diaphragmatic and suspensory ligament abnormalities. After the development of the main liver, the CL develops separately and temporally [10]. The retrohepatic vena cava along with the dorsal part of the liver originate as a common entity that is distinct from the rest of the liver. Moreover, the dorsal liver sector is evidently exclusive of the right and left liver lobes embryonically and anatomically [10]. Owing to the reformation of the liver in the postnatal period, the accessory fissures have the propensity to disappear while some may persist [3]. Moreover, accessory fissures in the QL were described in detail which has been performed by few studies to date [11]. In view of gross measurement, the midpoint of the hepatic part of the IVC proves to be an ideal reference point for the determination of the right margin of the CL as it stays unchanged despite the narrowing or widening of the IVC. The dilatation of the portal vein due to portal hypertension in cirrhosis renders it unsuitable for the aforementioned reference point [12].

Right and left lobes

In this study, the RL (72.5%) presented with substantially higher accessory fissures as compared to the LL (30%) located mainly on the visceral surface. Singh and Rabi observed 51.43% and 11.43% of right and left-sided fissures respectively and also with similar inferences on visceral findings [2].

Changes in the LL evident in Netter’s Types 2 and 4 are dependent on multiple factors including age, obesity, and coexistent splenomegaly. Following splenectomy, the left lobe may migrate and mimic the accessory spleen or a mass lesion around the porta hepatis [13]. Beaver’s lobe (Netter’s Type 4) has been reported by a few authors with incidences ranging from 1.81% to 12.86% as compared to 17.5% in our study. Livers with the deep renal impression (Netter’s Type 6) were reported by a few authors in lesser proportions ranging from 1.25% to 9.09% as compared to 7.5% in our study (Table 5).

**Table 5:** Comparison of morphological categories of various studies according to Netter’s classification - No value recorded

Authors	Mehare et al. [14]	Nagato et al. [15]	Patil et al. [16]	Sangeeta et al. [17]	Current study
Netter’s type 1	60.61%	52.46%	84%	66.9%	55%
Netter’s type 2	21.21%	8.19%	2%	7%	10%
Netter’s type 3	-	1.64%	-	3%	-
Netter’s type 4	6.06%	6.56%	10%	7.10%	17.5%
Netter’s type 5	3.03%	21.31%	2%	9%	2.5%
Netter’s type 6	9.09%	9.84%	2%	6%	7.5%
Netter’s type 7	-	-	-	-	7.5%

In this current study, Reidel’s lobe was observed in 2.5% as compared to 1.25% reported by Chaudhari et al. [5]. The congenital basis lies in a disembrioplastic anomaly in the growth of the hepatic bud which leads to the development of accessory lobes in infrahepatic positions. Owing to age, skeletal anomalies, and intraperitoneal or intrapelvic inflammation there is the possibility of hepatic modifications [18].

Diaphragmatic grooves (Zahn’s grooves) on the surface (Netter’s Type 7) have been recorded by two studies with varying incidences of 7.5% and 40% as compared to 7.5% in our study (Figure 4A) [5,19]. The sulci in the superior surface are mainly acquired and could be associated with obstructive pulmonary disease [3]. The appearance of sulci produced in the region of susceptible weak zones of superficial liver parenchyma is probably due to the pressure wielded by the diaphragm rather than the feat of hypertrophied muscles [19]. In the young, this pressure exerted might represent a hindrance to liver parenchyma’s homogenous growth at the level of watershed zones. Whereas in adults, a pathology may induce a chronic increased activity of diagrammatic pressure exerted by the muscle mainly at preformed weak zones [19]. Clinically, the diaphragmatic sulci characterize a beneficial landmark for the portal fissures and the superficial prediction of the deep course of the hepatic veins and their tributaries, representing morphological evidence of the functional vascular anatomy of the liver [19].

The gross measurements of the right and left lobes depicted in Table 4 have not been studied as much. The abnormal development of the RL could lead to portal hypertension whereas the LL maldevelopment might lead to gastric volvulus [20].

The determination of the ratio of the transverse diameter of CL to that of RL (CL/RL ratio) > 0.65 on imaging is considered sensitive and specific for the diagnosis of cirrhosis [21]. The range achieved in carrying out the CL/RL ratio in this study was 0.15-0.67 which was comparable to the two studies (Table 6) [12,22].

**Table 6:** Comparison of gross measurements (cm) of the external surface of the liver with other studies - No value recorded

Measurement categories		Reddy et al. [11]	Chavan and Wable [12]	Arora et al. [22]	Current study	
Length of the left lobe		-	-	-	7.8-27	
Width of the left lobe		-	-	-	4.6-10.5	
Length of the right lobe		-	6.7-10.5	5.29-9.93	10.2-26	
Width of the right lobe		-	-	-	6.7-11.5	
Length of the caudate lobe		2.4-7.8	4.0-9.3	3.38-7.03	3.3-9.3	
Transverse diameter (TD) of the caudate lobe		1.1-3.2	2.5-4.2	1.2-4.24	1.2-4.2	
Length of the quadrate lobe		2.38-9.1	-	-	2.8-10.3	
Transverse diameter of the quadrate lobe		1.57-6.3	-	-	2.5-5.2	
TD-Caudate lobe/TD-Right lobe (CL/RL) Ratio		-	0.28-0.46	0.15-0.58	0.15-0.67	

Interestingly, the radiologist may be the first to diagnose cirrhosis using the CL/RL ratio, if latent with no clinical or laboratory evidence. Clinically, liver cirrhosis is frequently accompanied by right lobe atrophy and hypertrophy of the left lobe’s lateral segment [23].

Caudate lobe

In this current study, the most common shape documented was rectangular (40%) followed by pear (pyriform) (12.5%) and inverted pyriform (10%) (Table 2). The most common shape reported by various authors is rectangular with incidences ranging from 48% to 94.5%. Arora et al. recorded a rare elongated CL in 2.77% as compared to 7.5% in our study [22]. Clinically, a particularly large-sized segment 1 (caudate lobe) is considered a diagnostic sign of cirrhosis [23]. The surgical anatomy of the CL is considered vital for a surgeon’s repertoire [10].

Reddy et al. described a prominent PP in 46.25% and a very prominent PP in 18.9% but in our study 32.5% and 17.5% respectively [11]. Chavan and Wabale observed the absence of PP in all livers whereas in our study it is 7.5% [12]. In this current study, prominent CP was found in 15%, just discernable in 7.5% whereas the rest were normal. Sarla et al. [24] observed prominent CP in 9% whereas Reddy et al. reported that all CP were easily discernable [12].

The PP may be mistaken for an extrahepatic pathology without adequate knowledge of sectional anatomy. Also, if the PP is affected by focal disease or its diffuse enlargement could enhance the possibility of CT misinterpretation [25].

Quadrate lobe

There is a dearth of literature on the QL as it hasn’t been the focus of studies, unlike the CL possibly owing to few clinical and surgical implications. The most common shape of QL in our study is quadrangular (25%), followed by triangular (17.5%), and rectangular (15%). Reddy et al. reported the most common shape of QL as quadrangular (85%) and rectangular (10%) [12]. PH was observed in 5% of our study, whereas in other studies, it varied between 1-30% (Table 7) [2,4,5,16,26,27].

**Table 7:** Comparison between different studies concerning morphological characteristics of the liver - No value recorded IVC: inferior vena cava

Morphological category		Singh and Rabi [2] (%)		Joshi et al. [4] (%)			Chaudhari et al. [5] (%)	Patil et al. [16] (%)		Nayak et al. [26] (%)	Muktyaz H et al. [27] (%)	Current study (%)
Reidel’s lobe		-		-			1.25	-		-	-	2.5%
Accessory lobe		12.86		-			3.7	2		-	-	5%
Pons hepatis- connecting Quadrate lobe with left lobe		22.86		30			1.25	10		-	-	5%
Pons hepatis- encapsulating IVC		-		-			-	-		-	-	2.5%
Accessory fissures overall		81.42		30			12.5	10		1.81	12.1	80%
Accessory fissure- Caudate lobe		27.14		-			3.7	-		-	-	57.5%
Accessory fissures -Quadrate lobe		-		-			-	-		-	-	17.5%
Absence of fissure for Ligamentum teres		-		-			11.2	4		1.81	9.7	-
Large papillary process		4.29		32			1.25	-		1.81	-	17.5%
Elongated left lobe		12.86		-			12.5	-		1.81	-	12.5%
Bilobed (Superior and inferior) Quadrate lobe		-		20			7.5	4		-	-	5%

The PH could obscure both the visualization of the fissure for ligamentum teres and the correct dimensions of the right and left lobes. One specimen (2.5%) was devoid of a QL in our study but Joshi et al. reported the same in 4% [4]. A small, buried, or absent quadrate lobe could induce errors and confusion in radiological diagnoses since the gall bladder fossa would be an approximation to the fissure for ligamentum teres. An accessory lobe is inherently dormant but could result in torsion, rupture, or even mimic acute abdomen [7]. Possible dislodgement of the primitive liver rudiment or mesodermal septa which persisted in proliferation could be the reason for moulding of the accessory liver tissue [28]. The QL is observed to atrophy in cirrhosis and may be due to portal venous hypoperfusion [23].

Accessory fissures

In this current study, accessory fissures were observed in 80% of livers, 57.5% of CLs, 17.5% of QLs, 72.5% of RLs, and 30% of LLs. Various studies about accessory fissures are compared in Table 7. Previously, these fissures have been stated to occur more commonly in the RLs followed by the QL which is not the observation in our study [2,4]. In the CL, 71.4% were vertical fissures in our study in comparison to 30-35% in other studies [11]. Rest was mainly transverse and oblique in nature. Reddy et al. accounted for 43.75% of fissures on CL, out of which 35% were vertical and 2.5% transverse. It has been stated that the RL presents with more sulci in line with sectors and segments. Few suggest that an unequal growth of the liver parenchyma may result in sulci whereas the other theory remarked that the fissures may be the marks of hepatic weak zones based on radiological and corrosion methods [3]. It is to be noted that accessory fissures not only are more common than accessory lobes but are significant clinically as they could be associated with organ dysfunctions such as diaphragmatic hernia, portal hypertension, and gastric volvulus [3]. Additionally, it could be mistaken for a lacerated liver in a patient with abdominal trauma [29].

The RL presented with vascular fissures/Rouviere’s sulcus/fissure of Gans (20% specimens) in our study which bears immense surgical significance in laparoscopic cholecystectomy or segmental resection as it aids in recognition of biliary pedicle [1]. This has only been reported by a few studies ranging between 4.7% to 27.3% [1,8]. Diagnostic imaging errors can be chiefly attributed to accessory fissures. On the other hand, they aid in planning hepatic resections. Any collection of fluid in these fissures may be mistaken for a liver cyst, liver abscess, hematoma, liver cyst, macronodular liver, or intrahepatic hematoma [2]. Implantation of peritoneally-disseminated tumor cells into these fissure spaces may imitate intrahepatic focal lesions [4].

Accessory notches

In this current study, 12.5% of livers were observed with accessory notches. Additional notches have been documented by Singh and Rabi in 10% of livers [2].

These variations in the morphological parameters in our study differ from the other studies may be due to population differences, as this study was conducted in the western part of India. 

Limitations of the study

The current study encompassed liver specimens, all acquired from donated bodies over the years. The limited number of specimens is a limitation owing to the nature of variations which could limit the generalizability of our observations to a bigger population. The authors propose a study in the future involving larger number of specimens leading to better understanding and representation of the variations.

## Conclusions

Few studies have ventured to document the liver variations in a wholesome manner encompassing almost all aspects. This current study has documented the variant shapes and accessory fissures on all four lobes which could pose difficulty in evaluating anatomical limits and pathology. The wide range of occurrence of morphological variations viz. detailed presence of Netter’s type livers, variable PP, PH, Reidel’s lobe, variable CP, and gross measurements will contribute to the evolving document of liver morphology and ultimately will be helpful for anatomists. Additionally, this may aid interventions and surgeons to plan hepatobiliary surgeries, liver transplantation, and maximize targeted approach and curtail confusion, thereby mitigating morbidity.
